# Resolving the tissue response to neoadjuvant chemotherapy in rectal cancer

**DOI:** 10.1016/j.xcrm.2023.101232

**Published:** 2023-10-17

**Authors:** Justin A. Shyer, Shannon J. Turley, Louis Vermeulen

**Affiliations:** 1 Genentech Inc, South San Francisco, CA, USA; 2 Cancer Center Amsterdam (CCA) & Oncode Institute, Amsterdam UMC, Amsterdam, the Netherlands

## Abstract

In this issue of *Cell Reports Medicine*, Qin et al.^1^ present a comprehensive single-cell transcriptomics analysis of the tumor microenvironment of rectal cancer tumors before and after neoadjuvant chemotherapy.

## Main text

Colorectal cancer (CRC) is the second most common cause of cancer-related deaths worldwide.^2^ Although colon cancer confined to the bowel and local lymph nodes can be effectively treated with surgery followed by adjuvant chemotherapy, in more advanced stages, rectal cancer (RC) remains an ongoing treatment challenge, primarily due to the anatomic location of the disease. The current mainstay of treatment for locally advanced RC is surgical resection preceded by neoadjuvant radiation or chemoradiation to reduce local recurrence. However, these therapies are often associated with significant morbidities, including urinary and sexual dysfunction and fecal incontinence. More recently, neoadjuvant therapy with chemotherapy consisting of a fluoropyrimidine (capecitabine or 5-FU) in combination with oxaliplatin has been shown to be effective in the neoadjuvant setting with similar rates of local control and the occurrence of distant metastases.^3^^,^^4^^,^^5^

The response to neoadjuvant chemotherapy (NAC) in RC is highly heterogeneous, ranging from rare progressive disease to complete pathologic responses. The ability to predict these responses to therapy would be of great value in determining the optimal treatment plan for individual patients. In addition, a better understanding of cellular responses in the context of a complex tissue architecture will be essential for the development of more effective therapies in this setting. To achieve this, a comprehensive high-resolution analysis of RC tissues consisting of both treatment-naive and post-treatment samples is essential. To date, such datasets have been lacking as they are technically and logistically very difficult to obtain. Therefore, the efforts described in this issue by Qin et al.^1^ to generate a highly valuable cohort need to be applauded.

**Figure 1:**
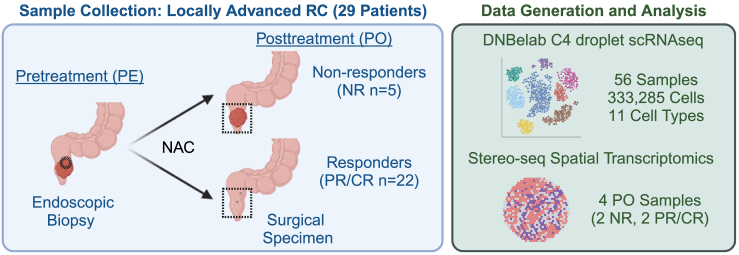
Schematic of study design in Qin et al. The authors collected pre- and post-treatment tumor samples from 29 patients treated with NAC. Samples were processed for scRNA-seq, with a subset also analyzed using spatial transcriptomics profiling. Created with BioRender.com.

Qin and colleagues use single-cell transcriptomics (scRNA-seq) and spatial transcriptomics profiling to define the cellular composition of the tumor microenvironment (TME) in 29 RC patients. Importantly, tumor samples were collected both before and after NAC without radiation treatment (Figure 1). These data provide a unique and rare insight into the tumor tissue response to NAC and the potential mechanisms that underlie response and resistance to chemotherapy treatment. Although previous studies have profiled human CRC samples using scRNA-seq in various contexts, most focus on specific cellular compartments and lack paired pre- and post-treatment samples.^6^ In contrast, the dataset presented by Qin and colleagues profiles stromal, immune, and epithelial cells from pre-treatment biopsies and post-treatment surgical resection samples, offering unprecedented insight into cellular heterogeneity and positioning, response to treatment, and association with clinical outcome.

A particular strength of the study is the clear clinical annotation that comes with the dataset, including patient and pathological characteristics, as well as details regarding the exact composition of the systemic therapy received, such as the duration of therapy and the clinical and pathological response. The presented data are not only valuable for RC but also for colon cancer given the biological similarities between these disease locations and the treatments.

In their manuscript, Qin and colleagues present several analyses that demonstrate the potential utility of the dataset. In one example analysis, they use correlations between clinical response and cancer-associated fibroblast (CAF) cell subtype abundance to propose potential roles of CAF subtypes in promoting the anti-tumor immune response and epithelial-mesenchymal transition following NAC. They demonstrate that CAF transcriptional phenotypes are significantly different between post-treatment samples of patients who exhibited complete tumor regression versus patients who did not respond to treatment, suggesting a role for CAFs in NAC treatment resistance. These findings are of particular interest and provide greater resolution to previous studies that used bulk sequencing to implicate genes, particularly those related to transforming growth factor-β (TGFβ) signaling in the tumor stroma, to poor outcomes in multiple CRC subtypes.^7^ Further analysis of the stromal compartment paired with mechanistic preclinical studies are warranted to better understand the disease-promoting and therapy-resistant mechanisms of RC CAFs.

In addition to scRNA-seq data, the study also consists of samples derived from four patients for which spatial transcriptomics was performed. Throughout the study, findings in the scRNA-seq are mapped onto the spatial maps to provide insight into interactions between various cell (sub)types. For example, different CAF subsets colocalize with distinct immune subsets in responders versus non-responders. However, these integrative analyses also demonstrate the inherent difficulties of this approach, as sample numbers in these types of studies so far have been limited and the power to draw definitive conclusions is therefore relatively low. This is particularly relevant in a highly heterogeneous disease such as CRC. Continued improvements in spatial technologies to bring down costs, as well as the development of standardized analytical methods for the integration of scRNA-seq with spatial omics, will be critical to fully realize the benefits of these technologies in the future.

Nonetheless, the data made available by Qin and colleagues in this issue of *Cell Reports Medicine* are a highly valuable resource and provide a roadmap for future study design wherein pre- and post-treatment tissue sampling is possible. As the standard-of-care treatment landscape for advanced RC evolves, with recently reported results suggesting a benefit of total neoadjuvant therapy over chemoradiotherapy and encouraging results of immune checkpoint therapy in mismatch-repair-deficient cases, follow-up studies will be critical to understand the cellular mechanisms underlying clinical response and resistance in the various subtypes and to discover potential targets for combination treatment.^8^^,^^9^^,^^10^

## References

[1] Qin P., Chen H., Wang Y., Huang L., Huang K., Xiao G., Han C., Hu J., Lin D., Wan X. (2023). Cancer-associated fibroblasts undergoing neoadjuvant chemotherapy suppress rectal cancer revealed by single-cell and spatial transcriptome profiling. Cell Reports Medicine.

[2] Sung H., Ferlay J., Siegel R.L., Laversanne M., Soerjomataram I., Jemal A., Bray F. (2021). Global Cancer Statistics 2020: GLOBOCAN Estimates of Incidence and Mortality Worldwide for 36 Cancers in 185 Countries. CA. Cancer J. Clin..

[3] Schrag D., Shi Q., Weiser M.R., Gollub M.J., Saltz L.B., Musher B.L., Goldberg J., Al Baghdadi T., Goodman K.A., McWilliams R.R. (2023). Preoperative Treatment of Locally Advanced Rectal Cancer. N. Engl. J. Med..

[4] Deng Y., Chi P., Lan P., Wang L., Chen W., Cui L., Chen D., Cao J., Wei H., Peng X. (2019). Neoadjuvant Modified FOLFOX6 With or Without Radiation Versus Fluorouracil Plus Radiation for Locally Advanced Rectal Cancer: Final Results of the Chinese FOWARC Trial. J. Clin. Oncol..

[5] Mei W.-J., Wang X.-Z., Li Y.-F., Sun Y.-M., Yang C.-K., Lin J.-Z., Wu Z.-G., Zhang R., Wang W., Li Y. (2023). Neoadjuvant Chemotherapy With CAPOX Versus Chemoradiation for Locally Advanced Rectal Cancer With Uninvolved Mesorectal Fascia (CONVERT): Initial Results of a Phase III Trial. Ann. Surg..

[6] Wen R., Zhou L., Peng Z., Fan H., Zhang T., Jia H., Gao X., Hao L., Lou Z., Cao F. (2023). Single-cell sequencing technology in colorectal cancer: a new technology to disclose the tumor heterogeneity and target precise treatment. Front. Immunol..

[7] Calon A., Lonardo E., Berenguer-Llergo A., Espinet E., Hernando-Momblona X., Iglesias M., Sevillano M., Palomo-Ponce S., Tauriello D.V.F., Byrom D. (2015). Stromal gene expression defines poor-prognosis subtypes in colorectal cancer. Nat. Genet..

[8] Conroy T., Bosset J.-F., Etienne P.-L., Rio E., François É., Mesgouez-Nebout N., Vendrely V., Artignan X., Bouché O., Gargot D. (2021). Neoadjuvant chemotherapy with FOLFIRINOX and preoperative chemoradiotherapy for patients with locally advanced rectal cancer (UNICANCER-PRODIGE 23): a multicentre, randomised, open-label, phase 3 trial. Lancet Oncol..

[9] Bahadoer R.R., Dijkstra E.A., van Etten B., Marijnen C.A.M., Putter H., Kranenbarg E.M.-K., Roodvoets A.G.H., Nagtegaal I.D., Beets-Tan R.G.H., Blomqvist L.K. (2021). Short-course radiotherapy followed by chemotherapy before total mesorectal excision (TME) versus preoperative chemoradiotherapy, TME, and optional adjuvant chemotherapy in locally advanced rectal cancer (RAPIDO): a randomised, open-label, phase 3 trial. Lancet Oncol..

[10] Cercek A., Lumish M., Sinopoli J., Weiss J., Shia J., Lamendola-Essel M., El Dika I.H., Segal N., Shcherba M., Sugarman R. (2022). PD-1 Blockade in Mismatch Repair–Deficient, Locally Advanced Rectal Cancer. N. Engl. J. Med..

